# Confocal laser endomicroscopy features of gastric antral vascular ectasia

**DOI:** 10.1055/a-2584-1623

**Published:** 2025-05-06

**Authors:** Ahamed A. Khalyfa, Navkiran Kaur Randhawa, Rahil Desai, Varshita Goduguchinta, Mahnoor Inamullah, Kamran Ayub

**Affiliations:** 1 21710 Gastroenterology, University of Iowa Health Care, Iowa City, United States; 2 160343 Gastroenterology, Augusta University Medical College of Georgia, Augusta, United States; 3 6203 Franciscan Health Olympia Fields, Olympia Fields, United States; 4 Southwest Gastroenterology, Oak Lawn, United States; 5 21431 Gastroenterology, Silver Cross Hospital, New Lenox, United States

Gastric antral vascular ectasia (GAVE), commonly referred to as “watermelon stomach” due to its characteristic endoscopic appearance, is a rare yet significant cause of gastrointestinal bleeding. GAVE is characterized by ectatic mucosal capillaries and fibrotic changes in the gastric antrum, typically arranged in longitudinal stripes. Clinically, GAVE presents with overt bleeding or chronic occult gastrointestinal bleeding, leading to iron deficiency anemia. Symptoms such as fatigue, melena, or hematemesis are frequently reported, depending on the chronicity and extent of blood loss 1 2 .

The differential diagnosis for GAVE includes conditions such as portal hypertensive gastropathy (PHG), severe gastritis, and other vascular lesions such as angiodysplasia and Dieulafoy’s lesion. Severe gastritis, particularly hemorrhagic or erosive types, may present with similar clinical features of gastrointestinal bleeding. However, endoscopic findings in gastritis typically reveal diffuse inflammation, erythema, and erosions rather than the characteristic longitudinal vascular patterns seen in GAVE. Histologically, GAVE shows fibrin thrombi and vascular ectasia, which are absent in gastritis and PHG. Distinguishing between these conditions is crucial for appropriate management, as the treatment strategies differ significantly 1 3 4 .

Confocal laser endomicroscopy (CLE) is an advanced imaging technology that enables real-time microscopic visualization of the mucosa at a cellular level (with a magnification of 1100×), without the need for biopsies. To the best of our knowledge, we report the first CLE evaluation and description of GAVE ( Video 1 ). We also demonstrate that many histological features of GAVE, such as ectatic capillaries ( Fig. 1 **a** and Fig. 1 **b** ) and fibrin thrombi ( Fig. 2 **a** and Fig. 2 **b** ), can be identified using CLE. This non-invasive approach enhances diagnostic accuracy, eliminates biopsy-related risks, and is particularly useful in patients requiring immediate evaluation or those at high risk for complications 5 .

Confocal laser endomicroscopy of gastric antral vascular ectasia.Video 1

**Fig. 1:**
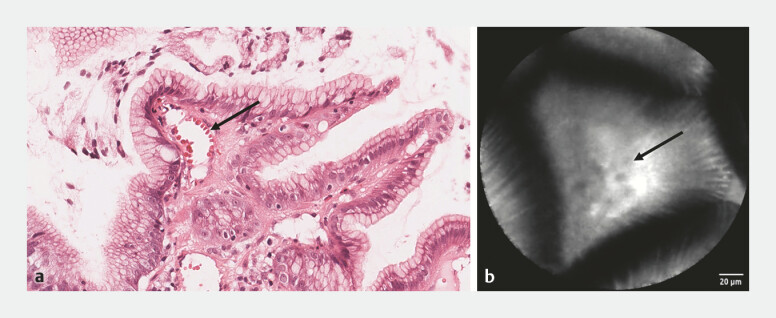
**a** Histopathology of GAVE showing dilated capillary with RBCs. Source: University of Leeds Virtual Pathology. **b** Confocal laser endomicroscopy of GAVE showing dilated capillary with RBCs. Abbreviations: GAVE, gastric antral vascular ectasia; RBCs, red blood cells.

**Fig. 2:**
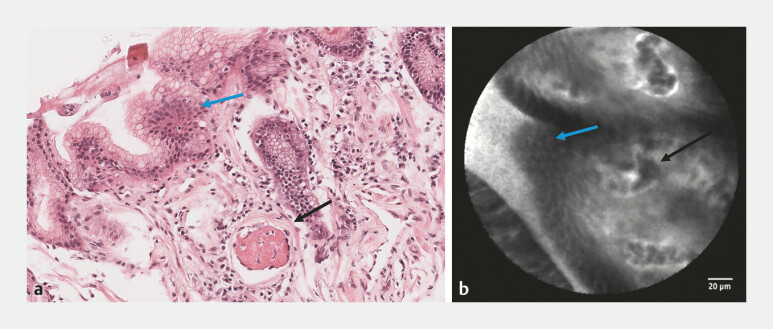
**a** Histopathology of GAVE with foveolar hyperplasia (blue arrow) and large intravascular fibrin clot (black arrow). Source: University of Leeds Virtual Pathology. **b** Confocal laser endomicroscopy of GAVE showing foveolar hyperplasia (blue arrow) and large intravascular fibrin clot. Abbreviation: GAVE, gastric antral vascular ectasia.

Endoscopy_UCTN_Code_CCL_1AB_2AD_3AZ

## Notes

Endoscopy E-Videos https://eref.thieme.de/e-videos
*E-Videos* is an open access online section of the journal *Endoscopy* , reporting on interesting cases and new techniques in gastroenterological endoscopy. All papers include a high-quality video and are published with a Creative Commons CC-BY license. Endoscopy E-Videos qualify for HINARI discounts and waivers and eligibility is automatically checked during the submission process. We grant 100% waivers to articles whose corresponding authors are based in Group A countries and 50% waivers to those who are based in Group B countries as classified by Research4Life (see: https://www.research4life.org/access/eligibility/ ). This section has its own submission website at https://mc.manuscriptcentral.com/e-videos .
